# Hypertensive disorders of pregnancy share common cfDNA methylation profiles

**DOI:** 10.1038/s41598-022-24348-6

**Published:** 2022-11-18

**Authors:** Marialuigia Spinelli, Jarmila A. Zdanowicz, Irene Keller, Pamela Nicholson, Luigi Raio, Sofia Amylidi-Mohr, Beatrice Mosimann, Daniel Surbek, Martin Mueller

**Affiliations:** 1 Department of Obstetrics and Gynecology, Department of Biomedical Research, University Hospital, Inselspital, University of Bern, Friedbuehlstrasse 19, 3010 Bern, Switzerland; 2 Department for BioMedical Research, Swiss Institute of Bioinformatics, University Hospital, University of Bern, Bern, Switzerland; 3 Next Generation Sequencing Platform, Institute of Genetics, Vetsuisse, University Hospital, University of Bern, Bern, Switzerland

**Keywords:** DNA methylation, Risk factors, Cardiology

## Abstract

Hypertensive disorders of pregnancy (HDP) contribute substantially to perinatal morbidity and mortality. Epigenetic changes point towards cardio-metabolic dysregulation for these vascular disorders. In early pregnancy, epigenetic changes using cell free DNA (cfDNA) are largely unexplored. We aimed to investigate these in HDP between 11 and 14 weeks of gestation by analysis of cfDNA methylation profiles in patients with hypertensive disorders. We identified patients without chronic hypertension but with subsequent development of preeclampsia (PE) (n = 11), with chronic hypertension (HT) but without PE development (n = 14), and lacking both PE and HT (n = 422). We matched patients according to PE risk factors into three groups (n = 5 each group): (1) PE: no HT but PE development, (2) HT: chronic hypertension but no PE and (3) Control: no PE or HT. We successfully optimized our cfDNA isolation process prior to whole genome bisulfite sequencing. Analysis of cfDNA methylation changes indicate a common predisposition in PE and HT groups, chiefly of maternal origin. Assessment of significant differentially methylated regions and annotated genes point towards a common cardiovascular predisposition in preeclampsia and hypertension groups in the first trimester. We postulate the pivotal role of the maternal cardiovascular system in HDP, which is already evident in the first trimester.

## Introduction

Hypertensive Disorders in Pregnancy (HDP) remain one of the most serious complications in pregnancy and include chronic (preexisting) and gestational hypertension as well as preeclampsia (PE)^1^. They contribute substantially to perinatal morbidity and mortality, but are also associated with an increased risk for cardiovascular disease for both mother and infant later in life^1,2^. While several maternal markers are being used and investigated to adequately predict outcome in pregnancies with HDP, the interplay between angiogenic, inflammatory and metabolic aspects on both fetal and maternal sides needs further investigation^3–5^. HDP are known to alter the hematological/immunological profiles and growth patterns of newborns and are also associated with subsequent high blood pressure and cardiovascular disease (CVD) risk in the offspring^6–8^. Along with the risk of intrauterine growth retardation/death or prematurity, the maternal phenotype consists of altered endothelial sensitivity and cardiovascular imbalance to placental factors^9–11^. The common maternal features and associations between HDP (in particular PE) and later CVD lead to an overarching theory of shared vascular predisposition, while epigenetic maternal memory seems to play a role as well^3,11^.

The genetic contribution to HDP based on inheritance patterns or mutations in candidate genes is established, but the potential contribution of epigenetic mechanisms only emerged recently^12,13^. Epigenetics does not involve changes in the DNA sequence but refers in part to the reversible addition of a methyl group (CH_3_) at the 5` carbon of a cytosine in a CpG dinucleotide context^8,11,14^. Therefore, DNA methylation patterns can change in response to stimuli such as age, environment, or diet^15^. In pregnancy, methylation patterns can change on multiple levels since both the placental/fetal and maternal compartment have to be considered. Initially, methylation patterns derived from the gametes are erased and a new profile is established in each individual^15^. This process means that the intrauterine milieu can affect fetal and offspring development^16,17^. For example, HDP alter DNA methylation patterns in the placenta and consequently in the offspring of poor placentation^12,18,19^. Besides placental/fetal modulation, the maternal compartment is exposed to epigenetic changes as well and in HDP these changes point towards cardio-metabolic alterations^3,20,21^. Not surprisingly, hypertension, ischemic heart disease, heart failure, cerebrovascular accident, and CVD based morbidities are more common in patients with recurrent PE^11^.

The emergence of cell-free DNA (cfDNA) in obstetrics paved the way for additional non-invasive assessments including HDP^22^. The first studies focused on detection of cfDNA levels or fractions (fetal and/or maternal cfDNA) as potential biomarkers of HDP^23–25^. Although the cfDNA fetal fraction is altered in women with subsequent PE development, the performance of PE screening was not significantly improved^26,27^. In contrast to cfDNA yield/fractions, the assessment of cfDNA methylation patterns potentially involves evaluation of both the cause and/or effect of HDP^22,28^. Recent systematic reviews summarized the role of DNA methylation in physiological and pathophysiological pregnancies including PE^29,30^. The majority of methylation studies conducted so far have focused on the placental compartment and mostly on selected candidate genes, emphasizing on outcomes and co-morbidities of pregnancy^30^. Reports on cfDNA genome-wide differential methylation regions (DMRs) or patterns, especially in early pregnancy, are absent^29^.

To address some of these gaps in knowledge, we postulate that the maternal cardiovascular system is more than just a victim of poor placentation. Hence, the aim of our study was to evaluate the shared cardiovascular hypothesis of HDP using the key role of cfDNA methylation in the first trimester^11,31^.

## Results

### Clinical outcomes

**Figure 1:**
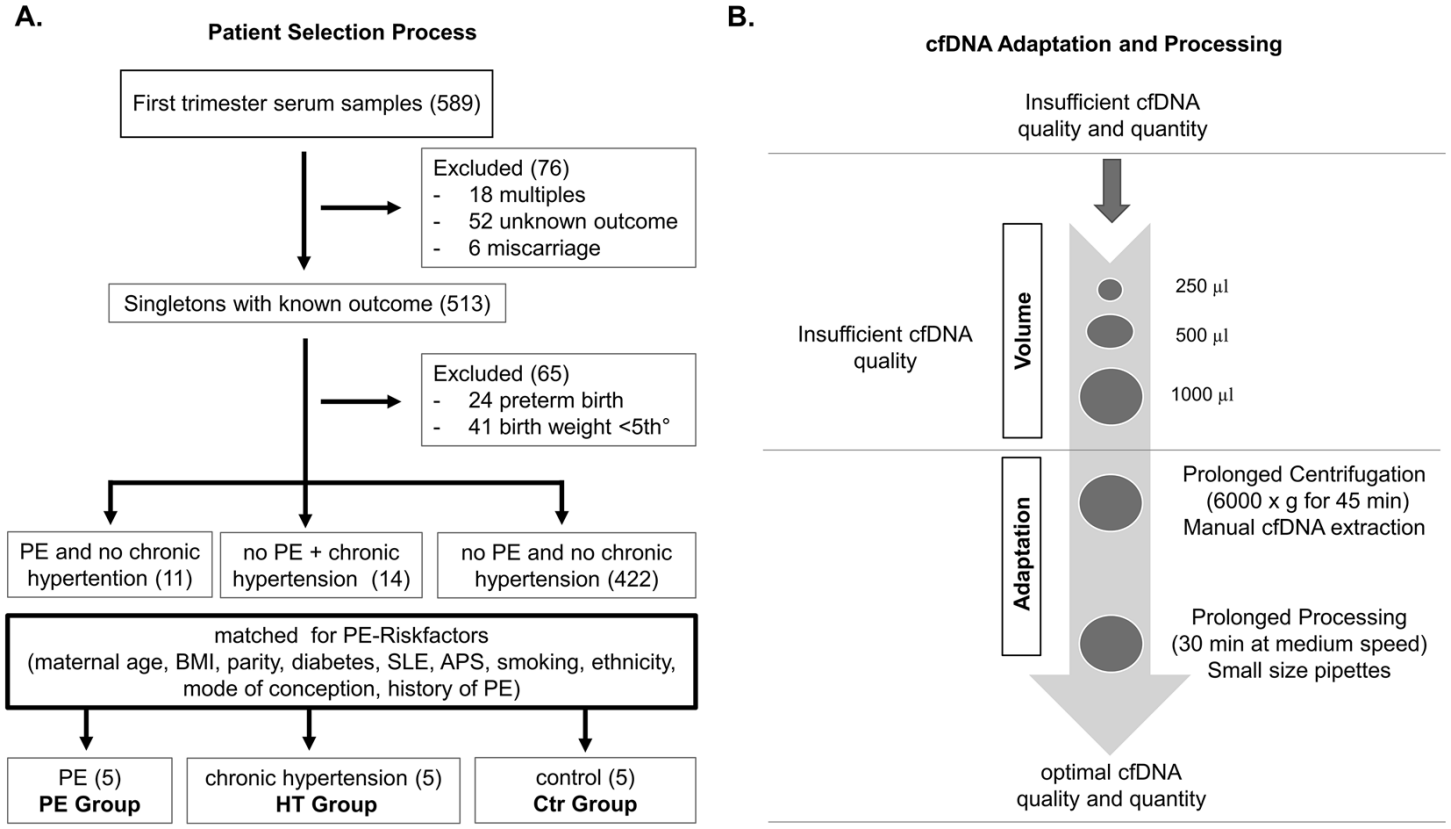
Patient and sample selection process. (**A**) Flowchart summarizing the selection process resulting in the final groups. (**B**) Flowchart summarizing the adaptation process to generate cfDNA of sufficient quality and quality. Sufficient cfDNA quality was assessed using the presence of the mono-nucleosome fragment around 170 bp, a di-nucleosome fragment around 350 bp, and a tri-nucleosome fragment between 550 to 580 bp. Preeclampsia (PE); Systemic Lupus erythematosus (SLE); Antiphospholipid Syndrome (APS).

Using a stringent selection, filtering, and matching process for our three study groups, we arrived at three study groups, each comprising of 5 patients (Fig. 1A). Namely, we established a preeclampsia group of selected patients who developed preeclampsia in the course of their pregnancy (PE) and matched this group with a chronic hypertension group made up of patients with chronic hypertension but no PE development (HT) and finally a control group comprising patients with neither PE nor chronic hypertension (Ctr). We decided to compare these three groups for the following reasons. We assumed that the presumably low-risk pregnancies but subsequently developing PE might have a common cardiovascular predisposition as the high-risk pregnancies with chronic hypertension. Given the preliminary nature of this study, we excluded patients with gestational hypertension as we speculated that these patients would have a lower number of cfDNA changes (in the first trimester) compared to patients with chronic hypertension.

As expected, the clinical characteristics across the selected groups differed only in the incidence of chronic hypertension and a positive result in PE screening (Table 1). Concerning perinatal outcomes across the three groups, we recorded significantly lower neonatal birth weight and percentile in the PE group compared to the HT or Ctr groups, which is in line with the expected poor placentation in patients developing PE (Table 2). The other outcomes such as gestational age at delivery, mode of delivery, or gestational diabetes incidence did not differ significantly between the groups.

**Table 1:** Clinical characteristics of patients for each cohort (n = 5 for each group), including parameters used for PE screening according to Fetal Medicine Foundation London^44^.

	PE group	HT group	Ctr group	*p* value
**Maternal characteristics**				
Maternal age (years), median [Interquartile range]	40 [35.0–45.5]	40 [31.0–43.0]	33 [32.0–37.5]	ns
Body mass index (kg/m^2^), median [Interquartile range]	29.3 [22.4–34.8]	31.8 [28.5–34.9]	29.1 [24.3–33.9]	ns
PE Screening positivity	2/5	5/5	0/5	< 0.05
**PE risk factors (positive)**				
Chronic hypertension	0/5	5/5	0/5	< 0.05
Smoking	0/0	0/0	0/0	_
Mode of conception	AIH: 2 IVF: 1 Spont: 2	IVF: 2 Spont: 3	IVF 2 Spont: 3	ns
Ethnicity (Caucasian)	5/5	5/5	5/5	_
Maternal history of PE	0/0	0/0	0/0	_
Previous pregnancies with PE	0/0	0/0	0/0	_
Type I or II diabetes	0/0	0/0	0/0	_
SLE	0/0	0/0	0/0	_
APS	0/0	0/0	0/0	_
Nulliparity	3/5	3/5	3/5	ns

*AIH* artificial insemination by husband, *APS* antiphospholipid syndrome; *Ctr* control, *IVF* in vitro fertilization, *HT* hypertension, *PE* preeclampsia, *SLE* systemic lupus erythematosus; *Spont* spontaneous conception.

**Table 2:** Perinatal outcomes (n = 5 for each group)**.**

	PE group	HT group	Ctr group	*p* value
**Maternal and neonatal outcomes**				
Mode of delivery	CS: 4 Vaginal: 1	CS: 4 Vaginal: 1	CS: 3 Vaginal: 2	ns
Gestational diabetes	2/5	2/5	1/5	ns
Gestational age median [range]	36.6 [29.1–40.7]	38.6 [36.9–41.0]	37.6 [37.1–40.6]	ns
Birth weight median [range]	2345 [945–3010]	3175 [2780–3950]	3285 [2850–3925]	< 0.05
Birth weight percentile median [range]	3 [1–66]	34 [21–81]	67 [28–81]	< 0.05

*CS* caesarean section, *Ctr* control, *HT* hypertension, *PE* Preeclampsia.

### cfDNA sample preparation

Once we identified the patients of interest, we focused on sample processing. Critically, our collected samples were professionally handled by the Liquid Biobank Bern to ensure correct handling and freezing procedures. Thereafter, we wanted to isolate cfDNA and perform whole genome bisulfite sequencing (WGBS). The application of cfDNA has proven successful in the field of obstetrics, especially in prenatal screening^32^. However, cfDNA isolation is not trivial, especially, with limited and precious starting material. Furthermore, the fragmented nature of cfDNA must be taken into consideration for any downstream application. As outlined in Fig. 1B, we had to extensively optimize the extraction process to recover sufficient cfDNA yield and integrity for the downstream application of WGBS. The gold standard approach for genome wide single base resolution evaluation of DNA methylation levels is WGBS and we were able to successfully execute this for all samples, enabling us to comprehensively investigate the shared cardiovascular hypothesis in HDP.

### Analysis of global DMRs changes indicates a common maternal predisposition of PE and chronic hypertension

**Figure 2:**
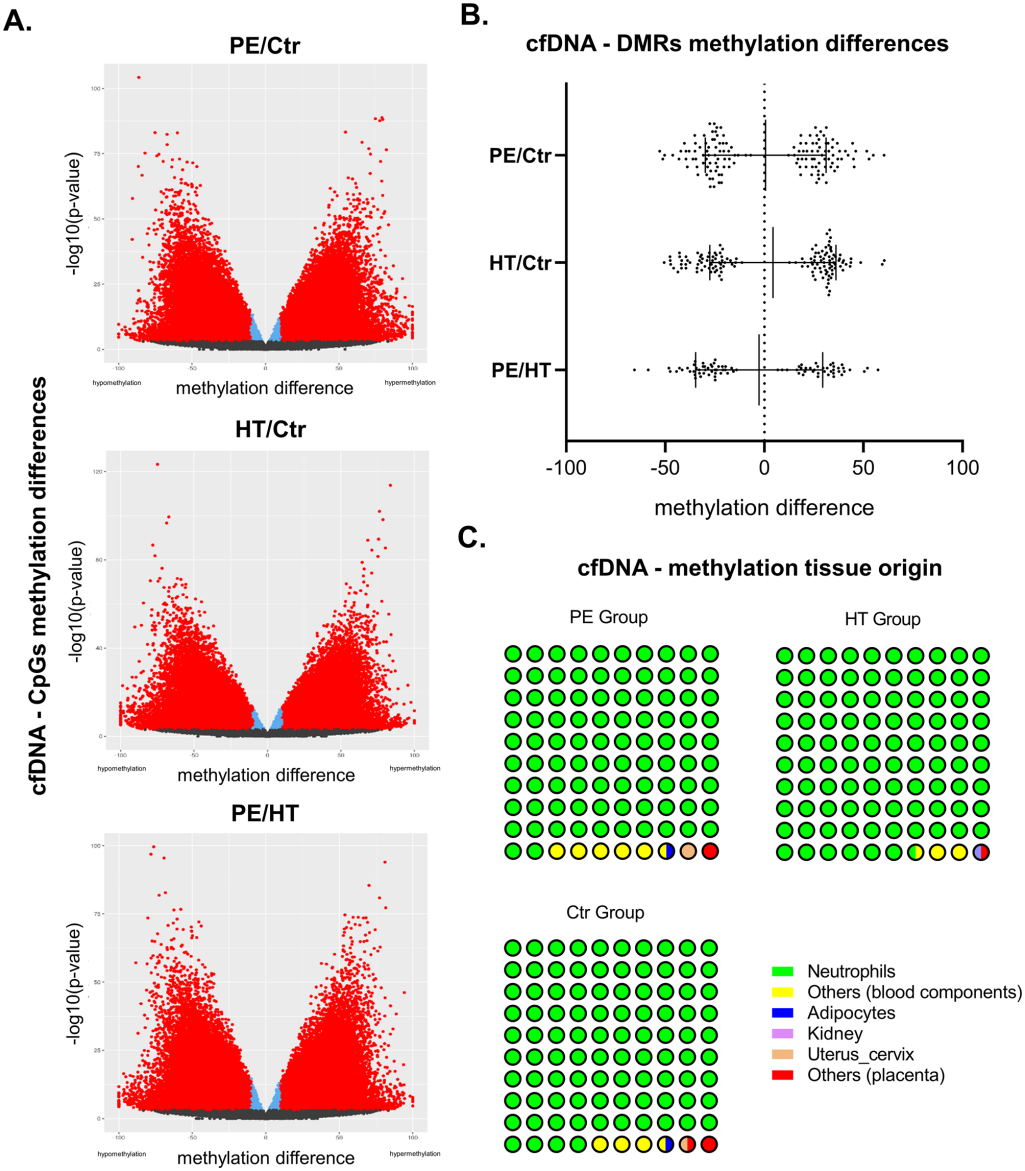
cfDNA methylation changes in the selected groups, n = 5 for each group. (**A**) Volcano plots visualizing the CpGs pairwise comparisons. On the *x*-axis is the difference in average methylation and *y*-axis the *p* value (-log10). All CpGs are shown as points: black—not significant; red or blue—significant (the adjusted *p* value is < 0.05). All CpGs shown in red have a methylation difference ≥ 10%. (**B**) Plots visualizing DMRs pairwise comparisons. On the *x*-axis is the pairwise comparison and *y*-axis the methylation difference. All DMRs are shown as points and differences significant (the adjusted *p* value is < 0.05). (**C**): Plots visualizing the predicted cfDNA source. Each dot symbolizes 1% and the legend summarizes the predicted methylation origin. Statistical processing was performed using GraphPad Prism and R Software, specifically the “ggplot2” package^58,62^. Differentially Methylated Regions (DMR); Preeclampsia (PE) Hypertension (HT); Control (Ctr).

Considering that cardiovascular system may not just be the victim of first trimester poor placentation, but eventually play a pivotal role in the pathogenesis of HDP, we tested global (fetal and maternal) cfDNA methylation profiles in first trimester serum samples^11^. We detected 133*′*515 CpGs (*5′—C—phosphate—G—3′)* differentially methylated between the PE/Ctr groups, 139*′*007 CpGs between the HT/Ctr groups, and 128*′*576 between the PE/HT groups (see Fig. 2A and Supplementary Table 1 for further details). No apparent or striking CpG methylation differences can be detected from the three volcano plots depicting CpGs pairwise comparisons shown in Fig. 2A. In a previous study, the combination of epigenome-wide high-throughput platforms and bioinformatics enabled the successful comparison of differentially methylated regions (DMRs) to detect associations in both physiological and pathological pregnancies (late gestational age)^29^. The DMRs assessment is a more precise tool to detect clinically relevant changes as opposed to CpGs and thus, we performed this analysis on our data. When assessing the DMRs pairwise comparisons PE/Ctr and HT/Ctr (Fig. 2B: compare PE/Ctr to HT/Ctr), we did not detect any apparent differences, which is expected in the first trimester^18,22^. Interestingly, the difference in the pairwise comparison PE/HT to both PE/Ctr and HT/Ctr is different and suggest a similar methylation profile of PE and HT samples (Fig. 2B: compare PE/HT to others). Notably, the PE group of patients were predominantly considered (and screened as) “low risk”, while the HT group as “high risk” at this time point using the FMF London PE Screening algorithm (Table 1). Leading on from this, we next wanted to evaluate the impact of the maternal and fetal compartments separately and thus, we performed unbiased determination of the tissue origins using deconvolution^33^. This statistical method predicts the potential cfDNA tissue origin. We detected only marginal placental cfDNA DMR methylation origin in all groups (Fig. 2C; compare red colored dots in all groups) suggesting that the detected cfDNA methylation changes likely originated from the maternal compartment. Therefore, the data collectively shows that the cfDNA methylation, alterations indicate profound changes in the maternal compartment in early pregnancy and that these changes suggest a similar predisposition/background in HDP. The next step was to evaluate the specific methylation signature and to this end, we performed DMRs specific gene annotations.

### Detailed DMRs changes point towards a common cardiovascular predisposition of PE and chronic hypertension

**Figure 3:**
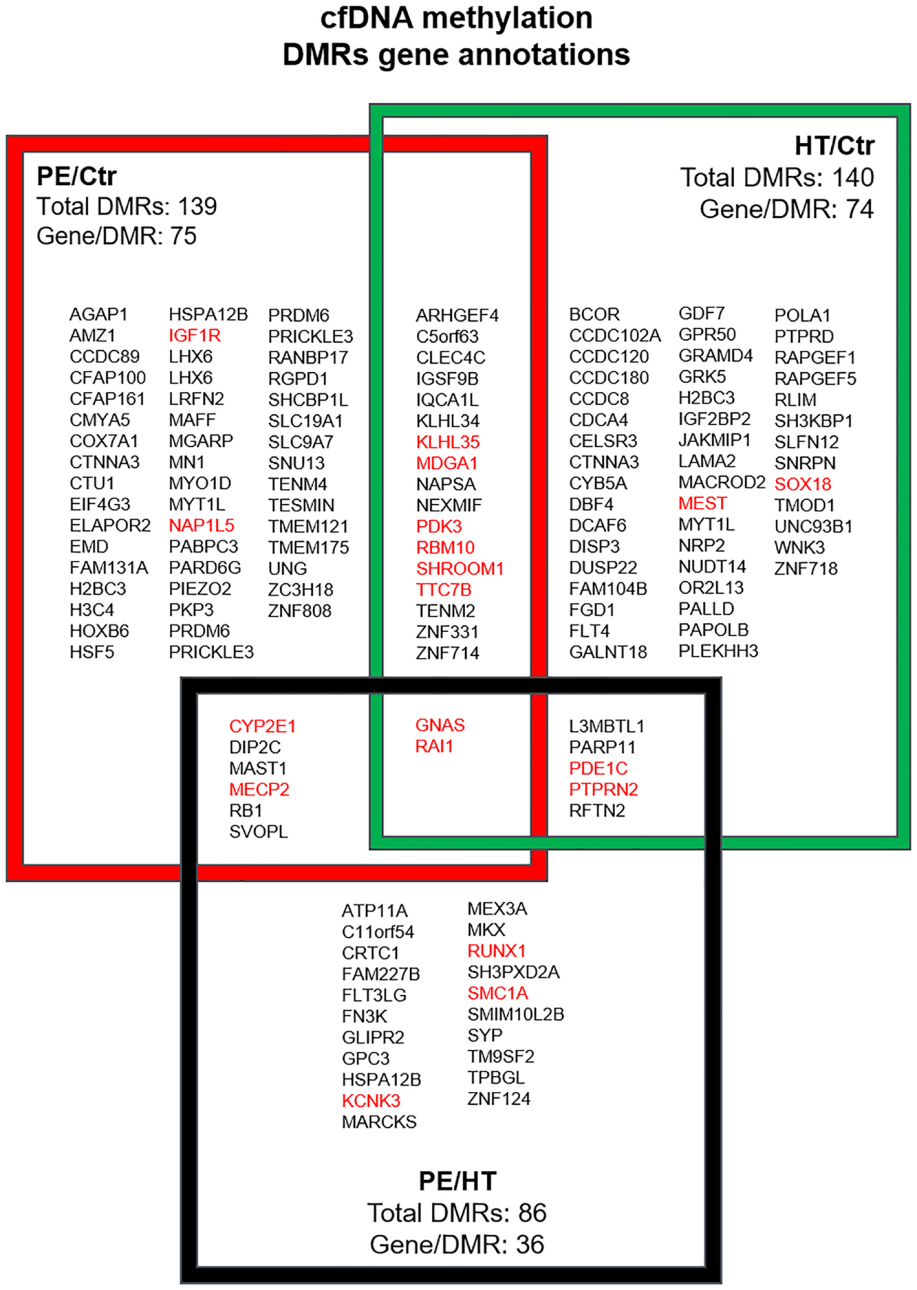
Detailed cfDNA methylation DMRs/Genes. We summarize the significant DMRs and annotated genes in each pairwise comparison (the adjusted *p* value is < 0.059 between all three groups (n = 5 for each group). PE/Ctr genes are bordered in red, the HT/Ctr in green and PE/HT in black. Annotated genes associated with cardiovascular disorders are highlighted in red. Differentially Methylated Regions (DMR); Preeclampsia (PE) Hypertension (HT); Control (Ctr).

Given the detected pairwise DMRs changes in the maternal compartment (Fig. 2B) and the theory of shared vascular predisposition, we aimed to shed light on maternal changes in the first trimester and analyzed genes methylation changes next^11^. Figure 3 details the significant DMRs and annotated genes in each pairwise comparison, PE/Ctr, HT/Ctr and PE/HT. We detected a lower number of DMRs (86) and genes (36) in PE/HT compared to PE/Ctr (139 DMRs and 75 genes) and HT/Ctr (140 DMRs and 74 genes). This lower number of DMRs and genes in PE/HT comparison and the relatively large number of DMRs overlapping between PE/Ctr and HT/Ctr comparisons (Fig. 3 compare overlapping boxes between PE/Ctr in red and HT/Ctr in green) point towards shared background/predisposition of PE and HT groups. Intriguingly, we found that many of the annotated genes to be associated with cardiovascular disorders (highlighted in red in Fig. 3). Taken together, cfDNA methylation profile assessment in the first trimester is possible and a non-invasive tool to predict maternal cardiovascular predisposition in the pathogenesis of HDP.

## Discussion

Our data suggests a shared predisposition to PE and chronic hypertension in our patient cohort, which we detected using cfDNA methylation profiles already in the first trimester^11^. We identified multiple DMRs and genes of interest pointing towards the association of HDP and cardiovascular disorders (Fig. 3 genes highlighted in red). Guanine Nucleotide binding protein Alpha Stimulating activity polypeptide (GNAS) appears to have a central role (Fig. 3) and is involved in several signaling pathways, such as regulation of glucose metabolism and blood pressure^34^. In line with this, methylation changes are associated with gestational diabetes or intrauterine growth restriction^35–37^. Furthermore, GNAS (contains DMR at 5' exons) is an imprinted gene and methylation changes correlate with transcript expression^38^. The importance of imprinted genes in HDP was reported, but emphasized only the importance of the fetal compartment^39^. Besides GNAS, we identified other imprinted genes of interest such as Zinc Finger Protein 331 (ZNF331) and Nucleosome Assembly Protein 1 Like 5 (NAP1L5), which are both associated with fetal growth control and cardiovascular development^40,41^. Another variant of ZNF named ZNF831 was identified using genome-wide association studies^42^. We did not detect changes of FLT1, which is associated with preeclampsia but identified a subtype called FLT4 in our hypertension and preeclampsia comparison^43^. Together, our data emphasizes the importance of imprinted genes in women at risk for developing HDP. Although multiple genome-wide association studies reported the associations previously, our non-invasive assessment in early pregnancy is novel. The separation of fetal and maternal compartments in cfDNA prior to whole genome bisulfite sequencing is ongoing and technically challenging.

Our results enable the possibility to develop new screening markers and algorithms. Currently, screening in early pregnancy incorporates established PE predictors such as maternal age, BMI, chronic hypertension, smoking, mode of conception, ethnicity, history of PE, previous pregnancies with PE, diabetes mellitus, SLE, APS, and parity. In addition to maternal characteristics, we incorporate biophysical markers such blood pressure and uterine artery Doppler and finally various biochemical markers in our routine screening to date^44^. Interestingly, of the ~ 160 biochemical markers described in literature for PE screening, only PAPP-A (pregnancy associated plasma protein A) and free β-human chorionic gonadotropin (β HCG) are established in the screening algorithm and both are of placental origin^45^. To date, aspirin prophylaxis in high-risk patients (stratified in the first trimester using FMF London algorithm) is the best approach to PE risk reduction^46^. General PE screening incorporates established PE predictors such as maternal characteristics (including chronic hypertension) and biophysical/biochemical markers, as described above^44^. Notably, chronic hypertension is a major risk factor for PE development, but these patients do not sufficiently benefit from aspirin prophylaxis^44,45^. In addition, only early onset PE has been shown to profit from aspirin prophylaxis, hence limiting this management of HDP disorders to a very specific group^47^. The use of the cfDNA fetal fraction to increase the performance of PE screening was not successful^27^. This is likely due to the substantial heterogeneity and complexity in the presentation and progression of PE and as well as the predominant use of placental markers^35^. HDP are vascular diseases presenting maternal altered endothelial sensitivity and cardiovascular imbalance to placental factors and thus the use of markers reflecting the maternal cardiovascular system is crucial^11^. This rather precision medicine approach to prophylaxis/treatment of HDP may include cfDNA methylation profiles as already developed in chronic cardiovascular diseases and oncology^36,48,49^.

We provide evidence that the maternal cardiovascular system in early pregnancy plays a pivotal role and contributes to the pathogenesis of HDP. This common cardiovascular predisposition of women with chronic hypertension and women with subsequent PE development underlines the role of pregnancy as “window to the future” identifying cardiovascular risks. Novel prophylaxis and treatment options can be envisioned, however, further understanding of the adaptation process is necessary. We imagine using cfDNA methylation as a part of the screening approach. If successful, the prophylaxis/treatment of cardiovascular maladaptation using already established drugs in cardiology such as statins, metformin, or sulfasalazine may be beneficial^50–53^. Novel drugs such as lipoprotein(a) lowering agents, colchicine or even a polypill approach can be imagined^54–56^. Here, we advocate the biological plausibility to test potential cardiovascular drugs in HDP. Such biological plausibility and safety of already approved drugs is essential prior to initiation of clinical trials in obstetrics. But also, as has recently been suggested, developing new drugs and treatments specifically targeted to HDP, based on an individual risk assessment should be explored^53^.

Our results demonstrate the need for further research into HDP, specifically the implementation as a valid screening method in early pregnancy as well as further differentiation of HDP, such as early and late onset preeclampsia or the inclusion of patients developing gestational hypertension. In addition, based on our screening, relevant treatment and prophylaxis can be used in the clinical routine, going beyond the use of aspirin to at risk patients, but using a tailored approach. A recent study emphasized the use of cell free material such as RNA to identify pregnancies at risk^57^. We postulate that the additional correlation with cfDNA, as presented here, would increase the potential of early prenatal testing.

One potential confounding factor in our analyses is the use of global (predominantly maternal) and not fraction (for example maternal and fetal) of cfDNA. Notably, the expected fraction of fetal cfDNA is ~ 10% and we predicted < 2% placental origin of the methylation changes (Fig. 2C)^26^. However, these changes are expected as we detect methylation changes and not cfDNA yield. We expect a tighter wrapping of DNA around nucleosomes and increase nucleosome compaction in the placental compartment^58^. Therefore, the change of cfDNA composition resulting in variation of accessibility will make the methylation changes in the placental compartment less evident at this stage of pregnancy^22^. This is in line with the analysis of specific genes, which suggest an association with the maternal cardiovascular adaptation process (Fig. 3). The major limitation of our study is the sample size of 15 patients, which has to be taken into account while interpreting the results. We included early and late-onset cases in the PE group to establish common methylation patterns in HDP in general. The differentiation between early and late-onset cases is important but beyond the scope of the manuscript. Importantly, we collected the samples in the first trimester (prior to aspirin prophylaxis) and the majority of the cfDNA methylation studies thus far was performed at later stages of the pregnancy or using placenta as origin.

The prevention and adequate treatment of long-term morbidities due to HDP require a personalized approach. We envision the use of cfDNA profiles in early pregnancy to predict the maternal cardiovascular system background risk and adaptation process in pregnancy.

## Methods

### Patient selection process

The patient selection process is depicted in Fig. 1. All patients were recruited in the first trimester (between 11 and 14 weeks of gestation; n = 589) and signed the consent form. This study was approved by Institutional Ethical Review Board (Cantonal Ethics Committee Bern, approval no. 2019-00431) and every procedure was conducted according to the Helsinki’s Declaration of Human Rights. Informed consent was obtained from all study participants.

Collected samples were stored in the Liquid Biobank Bern at − 80 °C, which guarantees a high quality and homogenous sample freezing process. Gestational age (GA) was initially calculated using the first day of the patient's last menstrual period and was later confirmed by an ultrasound examination. We defined HDP according to the guidelines of the International Society for the Study of Hypertension in Pregnancy (ISSHP)^59^. We excluded patients with multi-fetal gestation (n = 18), unknown outcome (n = 52), miscarriages (n = 6), fetal aneuploidy or lethal fetal anomalies, and associated preexistent serious maternal medical conditions, including preterm birth (n = 24) and small for gestational age (n = 41) apart from chronic essential hypertension. We excluded patients with birth weight < 5th percentile and normal blood pressure. Patient with blood pressure > 160 mmHg systolic or > 110 mmHg diastolic and at least one additional factor (according to ISSHP guidelines) including placental insufficiency were not excluded. We excluded patients with preterm birth, which were not related PE. Thereafter, three groups of patients were devised to evaluate the maternal compartment in HDP: 1. Patients without chronic hypertension but with subsequent PE development, 2. Patients with chronic hypertension but without subsequent PE development and 3: Patients comprising a control group since they did not have PE or chronic hypertension. We matched patients according to PE risk factors such as maternal age, body mass index (BMI), parity, preexisting diabetes, systemic lupus erythematosus (SLE), antiphospholipid syndrome (APS), smoking status, ethnicity, mode of conception, and personal/family history of PE. All patients were screened for PE between 11 and 14 weeks of gestation using the Fetal Medicine Foundation (FMF) London algorithm, which incorporates the aforementioned risk factors^44^. We considered a patient PE positive when presenting a risk calculation > 1:100 for PE below 37 weeks of gestation. The following parameters were recoded for each case: age, body mass index, smoking status, socioeconomic status, gravidity, parity, gestational age at birth, mode of delivery, further maternal or fetal pathological conditions, neonatal weight, neonatal APGAR at 1 and 5 min and potential neonatal complications. At the time of sampling, patients did not receive any medication that may affect the cfDNA methylation profiles such as antihypertensive drugs or aspirin. After blood sampling, patients screened positive received aspirin 150 mg 0-0-0-1 until 36 weeks of gestation.

### Cell-free DNA isolation

cfDNA was prepared from collected serum samples using a MagMAX Cell-Free DNA Isolation Kit (Thermo Fisher Scientific, A29319) along with a DynaMag-2 Magnet (Thermo Fisher Scientific, 12321D). We followed the accompanying user guide for the manual cfDNA isolation workflow (PN MAN0014327) but with several key modifications (Fig. 1B) to enable successful cfDNA isolation. Briefly, we started the protocol with 1000 µL of serum (250 µL and 500 µL did not yield successful results) and centrifuged the samples at 6000 × *g* for 45–60 min to remove any residual blood and cell debris. To lyse and bind the cfDNA to the beads, we vigorously mixed the serum, lysis/binding solution and magnetic beads by horizontal shaking at a medium speed for 30–40 min. After centrifugation, we placed the tubes on the magnet until the solution cleared and the beads were pulled against the magnet. We used a 10 µL pipette to fully discard the supernatant. During the ethanol washing steps, we tapped the magnet stand on the benchtop at least 10 times whilst keeping the sample tubes on the magnet. To elute the cfDNA, we mixed the solution by horizontal shaking at medium speed for 30–40 min and thereafter recovered the supernatant containing the purified cfDNA.

### Whole genome bisulfite sequencing (WGBS)

We assessed the quantity and quality of the extracted cfDNA using a Thermo Fisher Scientific Qubit 4.0 fluorometer with the Qubit dsDNA HS Assay Kit (Thermo Fisher Scientific, Q32854) and an Agilent Femto Pulse system with an Ultra Sensitivity NGS kit (Agilent, FP-1101), respectively. For the latter, we employed the following experimental parameters: an extended pre-run of − 3 kV; 2700 s, sample injection of − 5 kV, 30 s, separation voltage of − 1 kV, 240 min and no upper marker was used. Only samples displaying a mono-nucleosome fragment around 170 bp, a di-nucleosome fragment around 350 bp and a tri-nucleosome fragment between 550 to 580 bp, as well as larger cfDNA fragments were taken further. We generated Methyl-Seq libraries using an Accel-NGS Methyl-Seq DNA Library Kit and Unique Dual Indexing set (Swift Biosciences, 30,096 and 390,384, respectively). Briefly, we bisulfite converted between 8 and 50 ng of cfDNA using an EZ DNA Methylation Gold Kit (Zymo Research, D5005) without any prior fragmentation. Concerning cfDNA yield, we recorded the concentration between 0.25 and 2.5 ng/uL across groups and the overall distribution between all three groups was fairly similar. For the library preparation, we used samples ranging from 0.4 to 2.48 ng/uL using 20 uL of each samples (Supplementary Table 2). We used an average input of 22.7 ng/uL from 8 to 50 ng input material. All recovered DNA (10 µL) was used as input for library generation exactly following the protocol for cfDNA as an input and using SPRIselect beads (Beckmann Coulter, B23318) for clean-up steps. The quantity and quality of the final NGS libraries were assessed using a Thermo Fisher Scientific Qubit 4.0 fluorometer with the Qubit dsDNA HS Assay Kit (Thermo Fisher Scientific, Q32854) and an Agilent Fragment Analyzer (Agilent) with a HS NGS Fragment Kit (Agilent, DNF-474), respectively. The library pool was spiked with 10% PhiX Control v3 (Illumina, FC-110-3001) to compensate for reduced sequence diversity in bisulfite converted libraries and sequenced at 2 × 150 bp using a NovaSeq 6000 S4 Reagent Kit, 300 cycles (Illumina, 20,012,866) on the NovaSeq 6000 sequencing instrument operating NovaSeq Control Software v1.6. The run was assessed using Illumina Sequencing Analysis Viewer 2.4.7. Thereafter, Illumina bcl2fastq conversion software v2.20 demultiplexed sequencing data and converted generated base call files into FASTQ files. An average of 734 M reads were produced/sample. Next-generation sequencing of bisulfite-converted DNA to detect methylation status, from sample QC to sequencing run QC, was performed at the Next Generation Sequencing Platform, University of Bern, Switzerland.

### Statistical analyses

Clinical data handling and statistical processing was performed using Microsoft Excel, GraphPad Prism and R Software^60^. We used chi square tests for categorical data, Student’s *t*-tests and one-way analysis of variance (ANOVA) for normally distributed continuous data followed by the all pairwise multiple comparison Holm- Sidak test in case of a significant effect, and rank sum tests for non-normally distributed continuous data. We used linear and logistic regression to examine the influence of demographic and clinical data on the response variable. As standard, *p* < 0.05 was considered as statistically significant. For individual CpGs analysis, we used MethylKit. MethylKit allows an alternative way to analyze the data, which automatically applies a correction for overdispersion (i.e. variability that is larger than what the default model assumes). This approach is more robust and stringent than the previous analysis and produces far fewer significant CpGs (Supplementary Table 1: compare analysis without and with correction, as well as Supplementary Fig. 1). We employed nf-core methylseq v1.1 (https://nf-co.re/methylseq/1.1) for mapping the reads to the hg38 reference genome, marking duplicates, and extracting methylation calls with Bismark v. 0.22.2, and for producing a quality report. Thereafter, MethylKit v. 1.14.2 was utilized to test for differential methylation (DM) between pairs of experimental groups at the level of individual CpGs. The total number of CpGs considered in the MethylKit analysis is in the order of 26mio in all pairwise contrasts. Only CpGs with a minimum coverage of six reads in at least two samples per group were included. A CpG was differentially methylated if it had an FDR-adjusted P-value below 5% and a difference in average methylation between groups of at least 25%. For de novo identification of differentially methylated regions (DMR), we used metilene v. 0.2–8 and dmrseq v. 1.8.0^61^. Metilene is a specific tool to detect DMRs with a high sensitivity and specificity using stringent default settings. In particular, Bonferroni correction is applied to account for multiple testing. We retained all DMR with an FDR-adjusted *p* value below 5% in at least one of the two analyses. rGREAT v. 1.20.0 was applied to identify Gene Ontology terms associated with unusually many DMR. The annotatePeaks Perl script from Homer v. 4.11 was used to annotate DM CpGs and DMR. Further packages that were used for the R analysis were “GenomicRanges”, “tidyverse” and “ggplot2”^62–64^.

We used meth_atlas for the deconvolution of cell types in the different samples and a custom script to match the genomic positions of CpGs to the Illumina Array identifiers expected by meth_atlas^33^. The final analysis used a total of ca. 236 K CpGs and the reference atlas file provided on GitHub (https://github.com/nloyfer/meth_atlas).

We acknowledge potential cofounder as well as batch effects. Given the sample size of 15, we have decided not to perform further statistical analyses as it would lead to overfitting of the models.

## Supplementary Information

Supplementary Information.

## Data Availability

Data supporting the findings of this study are available from the corresponding author upon request. Data are not publicly available due to privacy reasons.
