# Supplementary material for: Hypertensive disorders of pregnancy share common cfDNA methylation profiles
Source: Sci Rep. 2022 Nov 18;12:19837. doi: 10.1038/s41598-022-24348-6 (PMC9674847; doi:10.1038/s41598-022-24348-6)

**Supplementary Material**

**Hypertensive disorders of pregnancy share common cfDNA methylation profiles**

Marialuigia SPINELLI^1+^, Jarmila A. ZDANOWICZ^1+^, Irene KELLER^2^, Pamela NICHOLSON^3^, Luigi RAIO^1^, Sofia AMYLIDI-MOHR^1^, Beatrice MOSIMANN^1^, Daniel SURBEK^1^, Martin MUELLER^1^*

^+^ these authors contributed equally.

**Supplementary Table 1**: Number of differentially methylated CpGs (p-adjusted < 0.05). Control (Ctr), hypertension (HT) and preeclampsia (PE).

| **Comparison** | **Previous analysis without correction for overdispersion** | **Re-analysis with correction for overdispersion** |
| --- | --- | --- |
| HT_Ctr | 139007 | 504 |
| HT_PE | 128576 | 496 |
| Ctr_PE | 133515 | 484 |

**Supplementary Table 2**: Overview of the cfDNA yield. We present the recovered and average input of the cfDNA. Control (Ctr), hypertension (HT) and preeclampsia (PE).

| Name | ng/µL |  | recovered cfDNA ng/µL |  | protol input | average input/group |
| --- | --- | --- | --- | --- | --- | --- |
| HT1 | 0.708 |  |  |  | 14.16 |  |
| HT2 | 2.48 |  |  |  | 49.60 |  |
| HT3 | 0.916 |  |  |  | 18.32 |  |
| HT4 | 1.32 |  |  |  | 26.40 |  |
| HT5 | 0.444 |  |  |  | 8.88 |  |
| **Average** |  |  | **1.1736** |  |  | **23.47** |
| **Range** |  |  | **0.4-2.48** |  |  |  |
| PE1 | 1.69 |  |  |  | 33.8 |  |
| PE2 | 1.37 |  |  |  | 27.4 |  |
| PE3 | 0.40 |  |  |  | 8.0 |  |
| PE4 | 1.08 |  |  |  | 21.6 |  |
| PE5 | 1.70 |  |  |  | 34.0 |  |
| **Average** |  |  | **1.248** |  |  | **24.96** |
| **Range** |  |  | **0.4 - 1.69** |  |  |  |
| Ctr1 | 0.496 |  |  |  | 9.92 |  |
| Ctr 2 | 1.15 |  |  |  | 23.0 |  |
| Ctr 3 | 0.958 |  |  |  | 19.16 |  |
| Ctr 4 | 1.32 |  |  |  | 26.40 |  |
| Ctr 5 | 0.992 |  |  |  | 19.84 |  |
| **Average** |  |  | **0.9832** |  |  | **22.7** |
| **Range** |  |  | **0.4 - 1.32** |  |  |  |

**Supplementary Figure 1**: Quantile-Quantile (Q-Q) plots comparing the three groups to each other. Plots using original data (A) and after overdispersion transformation (B). Control (Ctr), hypertension (HT) and preeclampsia (PE).

For statistical analysis, we used the “ggplot2” package^62^.


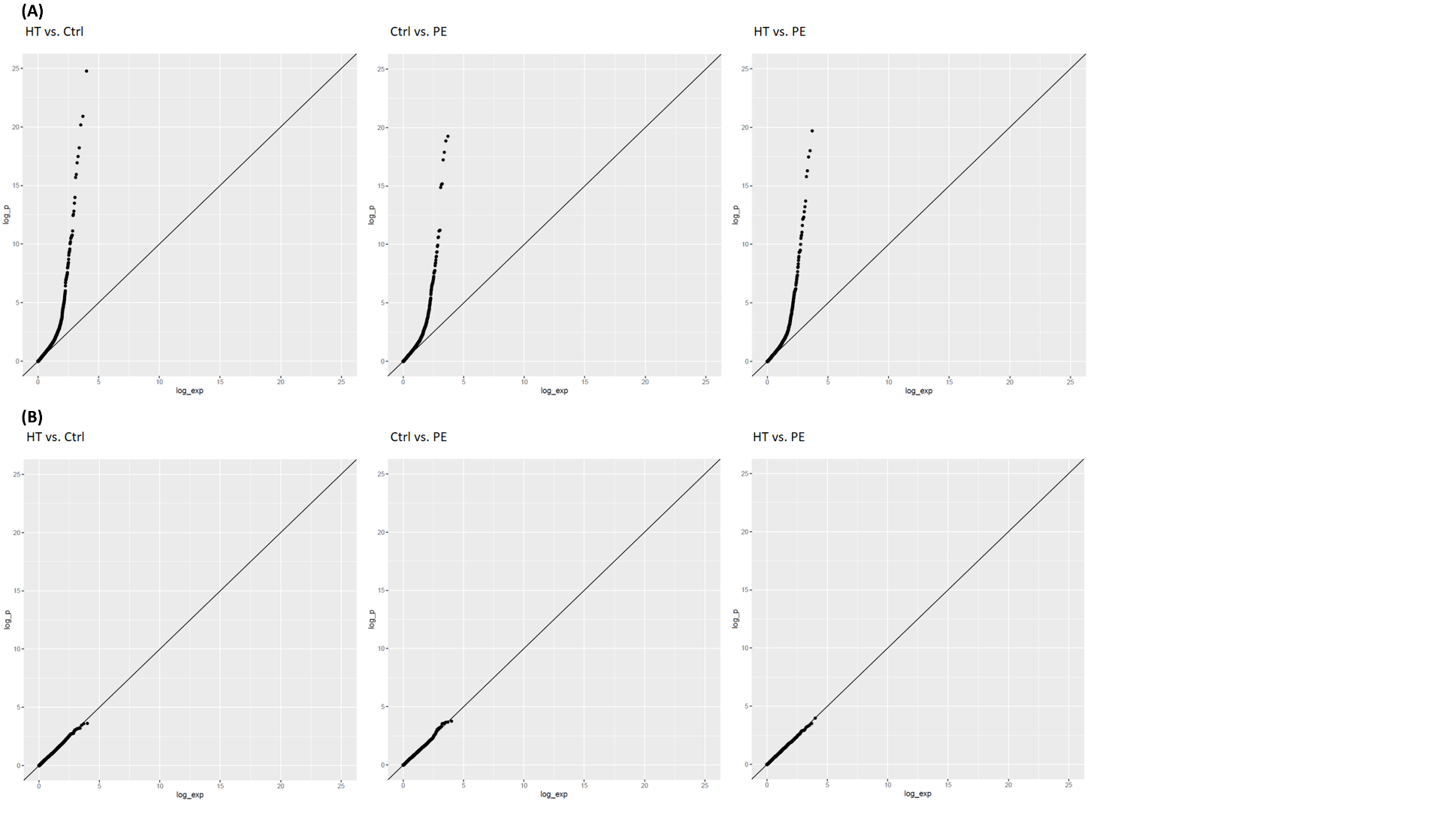

Supplement: Supplementary file 1 — Supplementary Information. [file 41598_2022_24348_MOESM1_ESM.docx]
